# Insights on the persistence of pines (*Pinus* species) in the Late Cretaceous and their increasing dominance in the Anthropocene

**DOI:** 10.1002/ece3.4499

**Published:** 2018-09-21

**Authors:** Surendra P. Singh, Inderjit, Jamuna S. Singh, Sudipto Majumdar, Jaime Moyano, Martin A. Nuñez, David M. Richardson

**Affiliations:** ^1^ Central Himalayan Environment Association (CHEA) Nainital India; ^2^ Department of Environmental Studies Centre for Environmental Management of Degraded Ecosystems (CEMDE) University of Delhi Delhi India; ^3^ Department of Botany Banaras Hindu University Varanasi India; ^4^ Grupo de Ecologia de Invasiones INIBIOMA CONICET/Universidad Nacional del Comahue Bariloche Argentina; ^5^ Department of Botany and Zoology Centre for Invasion Biology Stellenbosch University Matieland South Africa

**Keywords:** adaptations, biological invasions, *Diploxylon* pines, fire, *Haploxylon* pines, stressful environments, traits, tree invasions

## Abstract

Although gymnosperms were nearly swept away by the rise of the angiosperms in the Late Cretaceous, conifers, and pines (*Pinus* species) in particular, survived and regained their dominance in some habitats. Diversification of pines into fire‐avoiding (subgenus *Haploxylon*) and fire‐adapted (subgenus *Diploxylon*) species occurred in response to abiotic and biotic factors in the Late Cretaceous such as competition with emerging angiosperms and changing fire regimes. Adaptations/traits that evolved in response to angiosperm‐fuelled fire regimes and stressful environments in the Late Cretaceous were key to pine success and are also contributing to a new “pine rise” in some areas in the Anthropocene. Human‐mediated activities exert both positive and negative impacts of range size and expansion and invasions of pines. Large‐scale afforestation with pines, human‐mediated changes to fire regimes, and other ecosystem processes are other contributing factors. We discuss traits that evolved in response to angiosperm‐mediated fires and stressful environments in the Cretaceous and that continue to contribute to pine persistence and dominance and the numerous ways in which human activities favor pines.

## 1 INTRODUCTION

**Figure 1:**
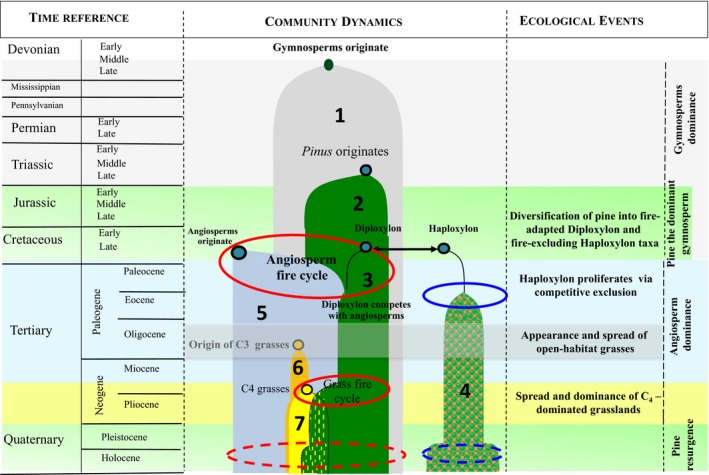
Key drivers and responses during the evolution of pines. Gymnosperms originated in the Devonian (350 Myr ago), but pines originated ~150 Myr ago. Oval shapes indicate environmental filters, red ovals for *Diploxylon* and blue ovals for *Haploxylon* pines. The presence of different groups is represented by numbers: 1, gymnosperms; 2, pines prior to diversification; 3, *Diploxylon*; 4, *Haploxylon;* 5, angiosperms; 6, C_3_ grasses; 7, C_4_ grasses. Solid red ovals indicate fire as a driver, and solid blue ovals denote extreme environments as drivers. Dashed red or blue ovals indicate the impact of humans. Angiosperms, mainly shrubs and herbs, appeared as understorey ruderals in the Late Cretaceous (65–145 Myr ago) and later proliferated in response to novel fire regimes. This resulted in the shift of community structure as slow‐growing gymnosperms were replaced by fast‐growing angiosperms, resulting in the decline of gymnosperms except conifers. Changes in the climate in the Miocene (5–24 Myr ago) led to the replacement of woodlands with grasslands dominated by C_4_ species. Highly flammable C_4_ grasses became abundant in open areas with higher light availability. *Pinus* has shown remarkable adaptability to highly flammable ecosystems, including frequently burned C_4_ grasslands. The timing of angiosperm evolution overlaps with the origin and diversification of pines into *Diploxylon* and *Haploxylon* taxa. *Haploxylon* taxa are fire‐avoiding and occur in drier and colder regions, whereas *Diploxylon* taxa are fire‐adapted and occur in more productive environments at subtropical and temperate latitudes. *Diploxylon* pines evolved to adapt to fire and codominate with angiosperms in some more productive regions outside tropical rain forests

Gymnosperms emerged in the Devonian (about 350 Myr ago), long before the appearance of angiosperms (Augusto, Davies, Delzon, & Schrijver, 2014) (Figure 1). The rise of the angiosperms and the concomitant decline of gymnosperms in the Late Cretaceous (about 100–60 Myr ago) are among the most important phytogeographic phenomena in the history of the planet. The radiation of angiosperms during the Cretaceous (termed an “abominable mystery” by Charles Darwin; Berendse & Scheffer, 2009) resulted in extraordinarily high species numbers and considerable expansion of the area occupied by angiosperms. Angiosperms attained dominance at low latitudes in the Late Cretaceous, while many gymnosperms (Bennettitales and Cheirolepidiaceae) went extinct (Bond & Scott, 2010). The emergence of angiosperms was more successful in suppressing nonconiferous gymnosperms such as cycads, seed ferns, and free‐sporing plant groups including mosses, lycopods, sphenophyta, ferns, and fern allies (Lidgard & Crane, 1988). Some conifers also declined during the ecological rise of angiosperms. Taxa in the extinct family Cheirolepidiaceae declined, and other conifers faced major species turnover during Late Cretaceous (Augusto et al., 2014). In general, however, modern conifers fared much better than other gymnosperms during the rise of angiosperms (Willis & McElwain, 2002); they are thus a poor proxy for gymnosperm lineages that were eliminated by angiosperms (Augusto et al., 2014).

**Figure 2:**
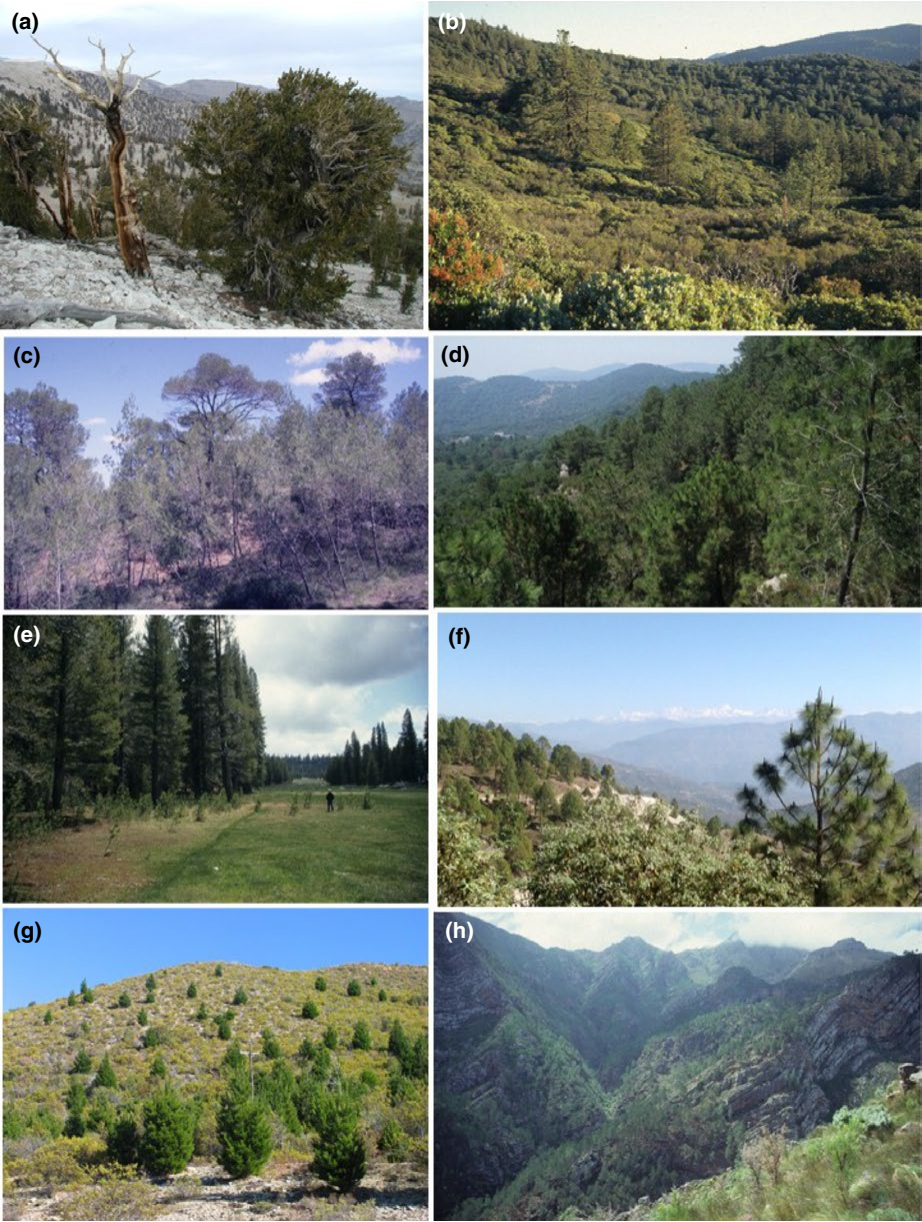
Pines in the Northern (native) and Southern (nonnative) Hemispheres. (a) *Pinus longaeva* in the White Mountains, California, USA; (b) *P. contorta*, southern California, USA; (c) *P. halepensis*, southern France; (d) *P. pinaster*, Andalucía, Spain; (e) *P. contorta*, colonizing subalpine meadow, Sequoia National Forest, California, USA; (f) *P. roxburghii* in the Himalaya, Uttarakhand, India; (g) *P. contorta* invading Patagonian steppe vegetation, near Bariloche, Argentina; (h) *P. pinaster* invading fynbos shrubland, Western Cape, South Africa. Photograph credits: D.M. Richardson (a–e, g, and h), Inderjit (f)

In response to competition posed by the emergence of angiosperms and changing fire regimes, pines (Figure 2) diversified into two lineages: (a) *Diploxylon* (fire‐adapted “hard pines”; subgenus *Pinus*) and (b) *Haploxylon* (“soft pines”; subgenus *Strobus*, which are fire avoiders) (López, Kamiya, & Harada, 2002). Fire‐avoider pines expanded into abiotic stressful environments, and fire‐adapted pines occurred in relatively productive environments of subtropical and temperate latitudes (Keeley, 2012; Schwilk & Ackerly, 2001). The large size of pines and the physiological and anatomical adaptations of (especially *Haploxylon*) pines helped them persist in extreme environments (Tomback & Linhart, 1990). *Pinus* (≈115 species), all but one species (*P. merkusii*) with native ranges confined to the Northern Hemisphere, is the coniferous genus with the largest number of species and the largest global distribution (Brodribb, Pittermann, & Coomes, 2012; Richardson, 1998). Pines had colonized the entire supercontinent of Laurasia by the end of the Cretaceous (Gallien, Saladin, Boucher, Richardson, & Zimmermann, 2016; Keeley, 2012). They currently occupy large areas in both temperate and subtropical ecosystems (Figure 2). The tropical distribution of pines is also associated with savannas in the southeastern United States (up to 12° latitude N), the Caribbean region, and parts of Central America.

Pines originated in the circumpolar continent of Beringia during the Mesozoic era and started expanding across lower latitudes around late Mesozoic and early Cenozoic eras (Mirov, 1967). Pine expansion aided the diversification in major extant clades of pines with Eurasian and North American origin through the Oligocene with various events of vicariance (Eckert & Hall, 2006). Palynological fossil evidence suggests a shift in dominance from broad‐leaved deciduous forest to pine‐dominated forest during the Pliocene and Pleistocene in eastern Europe (Stuchlik, 1994).

The Holocene, the current geological epoch, began approximately 11,650 cal years before present, after the last glacial period. The increase in human populations during this epoch and of the human footprint on the planet's ecosystems has led to calls for the Holocene to be seen as the first stage of the Anthropocene, or indeed for the terms “Holocene” and “Anthropocene” to be considered as synonymous (Certini & Scalenghe, 2015). In this paper, we use the term “Anthropocene” to refer to the period during which human activity has become the dominant influence on climate and the environment. Many pine species have undergone major range expansions in the Anthropocene by expanding their ranges in the Northern Hemisphere (Améztegui, Brotons, & Coll, 2010; Jakubos & Romme, 1993; Lubetkin, Westerling, & Kueppers, 2017; Prévosto, Hill, & Coquillard, 2003; Taylor, Maxwell, Pauchard, Nuñez, & Rew, 2016b; Taylor et al., 2016a) and, due to large‐scale plantings and invasions, mainly in the Southern Hemisphere (Essl, Mang, Dullinger, Moser, & Hulme, 2011; Richardson, Williams, & Hobbs, 1994; Simberloff et al., 2010). Pine is the dominant taxon in natural forests over parts of the Northern Hemisphere (Richardson, 1998), and more than 20% of pine species are invasive in regions outside their native ranges (Nuñez et al., 2017; Rejmánek & Richardson, 2013; Richardson & Rejmánek, 2004; Rundel, Dickie, & Richardson, 2014; Simberloff et al., 2010). Pines have expanded their ranges in the Northern Hemisphere due to planting by humans (e.g., Brundu & Richardson, 2016) and by encroaching into previously treeless ecosystems. Examples in North America are where the native species *P. contorta* has colonized grasslands and shrublands (Figure 2e; Jakubos & Romme, 1993; Lubetkin et al., 2017; Taylor et al., 2016b), in France where *P. sylvestris* has spread into abandoned lawns and heathlands (Prévosto et al., 2003), and in Spain where *P. uncinata* has expanded its range in the Pyrenees (Améztegui et al., 2010). Pine invasiveness is associated with ruderal strategies, large niche breadth, human use, historical biogeography, and climate (Essl et al., 2011; Gallien et al., 2016; Grotkopp et al., 2002; McGregor, Watt, Hulme, & Duncan, 2012; Pauchard et al., 2016; Procheş, Wilson, Richardson, & Rejmánek, 2012). Pine invasions are increasing in importance especially in areas with large‐scale afforestation with nonnative pines (Richardson, van Wilgen, & Nuñez, 2008). This process is evident in countries in the Southern Hemisphere such as New Zealand (Ledgard, 2004), South Africa (Figure 2h; Rouget, Richardson, Milton, & Polakow, 2001; Van Wilgen & Richardson, 2012), Argentina (Figure 2g; Nuñez, Horton, & Simberloff, 2009; Sarasola, Rusch, Schlichter, & Ghersa, 2006), Chile (Langdon, Pauchard, & Aguayo, 2010), and Brazil (Zenni & Simberloff, 2013). Several pine species are also invasive in the Northern Hemisphere where nonnative pines were introduced for forestry, for example, in Sweden (Engelmark et al., 2001) and the British Isles (McGregor et al., 2012).

**Figure 3:**
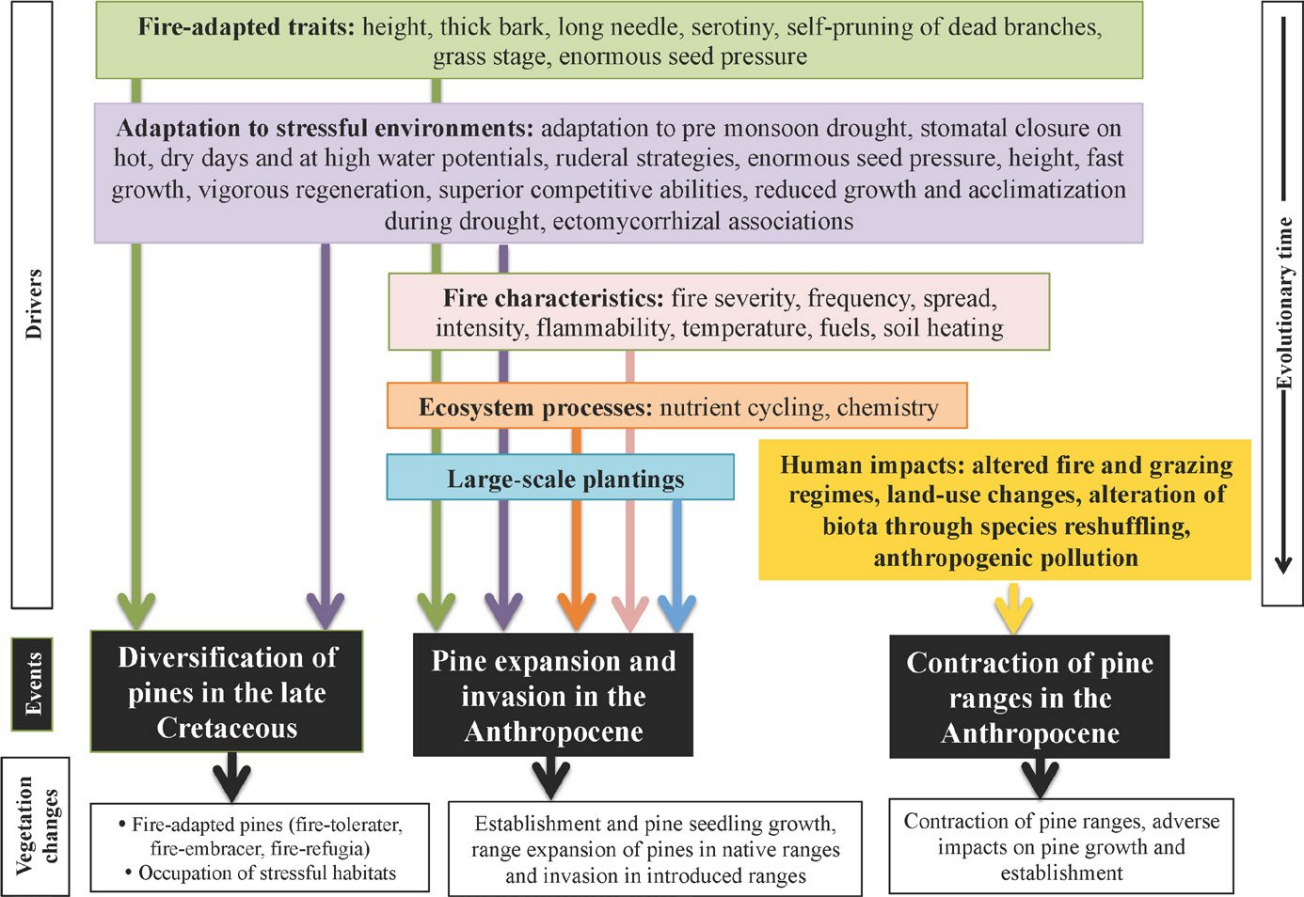
Schematic representation that connects the diversification of pines into two lineages (*Haploxylon* and *Diploxylon* taxa) to their diversification and domination over evolutionary time and their ability to (a) evolve novel traits and adaptations for extreme environments; and (b) manipulate nutrient cycling to dominate globally. It depicts the ecological drivers, traits, and responses during the evolution of conifers (pines) and angiosperms in evolutionary time. Drivers (colored boxes) of pine dispersal and dominance in evolutionary time (black boxes) resulted in the occupation of extreme environments by pines, coexistence of fire‐adapted pines with angiosperms in productive habitats, and expansion, invasion and contraction of pines in Anthropocene (white boxes). Fire regimes, life‐history traits, adaptations, and ectomycorrhizal associations played important roles in the diversification of pines into fire‐avoiding (*Haploxylon*) and fire‐adapted (*Diploxylon*) taxa in response to the emergence of angiosperms in the Late Cretaceous. As a result, fire‐avoiding pines occupied stressful extreme environments, which were much less suitable for angiosperms. Fire‐adapted pines share some productive habitats with angiosperms. Some pines are currently expanding their ranges in the Northern Hemisphere, and widespread plantings and associated human activities have resulted in widespread pine invasions in the Southern Hemisphere. Adaptations/traits that evolved in response to angiosperm‐mediated fire regimes and stressful environments in the Late Cretaceous were key to pine success through Neogene and Paleogene and also contribute to the pine dominance in the Anthropocene. For example, life‐history traits that were important in the pine diversification also are key driver of pine dominance in the Anthropocene. Other crucial drivers of pine dominance are as follows: ecosystem‐driven processes, fire as disturbance, and human‐assisted plantations. Human impacts on fire regime, grazing/browsing, use of pine material for construction purposes, manipulation of natural ecosystem, alteration of biota through species reshuffling, anthropogenic pollution and land use result in the adverse effects of the establishment, and growth and range size of pines

The radiation and emergence of angiosperms in the Late Cretaceous are well covered in the literature (Augusto et al., 2014). However, more work is needed to understand the diversification and dominance of pines in evolutionary time. We discuss two questions: (a) How pines could persist in the Late Cretaceous when angiosperms were emerging and spreading? and (b) Why have pines become the dominant conifer taxon in many environments over evolutionary time? The major events in pine diversification and dominance can be compartmentalized into three phases: (a) pine diversification in the Late Cretaceous; (b) current range expansion and invasion of pines; and (c) range contraction of pines. Five drivers play key roles in these phases: (a) fire‐adapted traits; (b) adaptations to extreme environments; (c) fire characteristics; (d) ecosystem‐mediated processes; and (e) large‐scale planting and dissemination by humans (Figure 3). There are similarities in the drivers of pine diversification in the Late Cretaceous and of pine dominance in the Anthropocene. Elaboration of these factors provides the means to unravel the causes that explicitly drive pine dominance (fire as an ecological disturbance, ecosystem‐mediated processes, and large‐scale pine planting by humans) (Figure 3). Such insights could be utilized to predict and manage pine forests in the future.

## 2 WHY PINES PERSISTED DURING THE RISE OF ANGIOSPERMS?

Conifers were widespread during the Cretaceous (Harland, Francis, Brentnall, & Beerling, 2007; He, Pausas, Belcher, Schwilk, & Lamont, 2012). The decline of gymnosperms happened in the Late Cretaceous when angiosperms were emerging. The adaptations of pines to fire regimes and the traits that allowed them to survive in extreme environments lead to the diversification of pines (Bond & Scott, 2010; He et al., 2012). In the following sections, we discuss adaptations to fire and traits that helped pines to survive in extreme environments in the Late Cretaceous.

### 2.1 Pine adaptations to fire regimes

Fire was a major driver of the rise of angiosperms in the Late Cretaceous (Figure 1; Bond & Scott, 2010). The timing of angiosperm evolution overlaps with the diversification of pines (Keeley, 2012), partly because angiosperms altered fire regimes by promoting more frequent fires than the slower growing gymnosperms. Pine therefore diversified into fire‐adapted and fire‐avoider lineages. In discussing fire‐adapted pines, Keeley (2012) identified pines with fire‐tolerater, fire‐embracer, and fire‐refugia strategies. Taxa with fire‐tolerater strategies have long needles and thick bark (Keeley, 2012). The thick bark of these pines protects their cambium from extreme heat due to intense fire, but their long needles contribute to intense fires by creating a loosely packed litter layer (Fonda, Belanger, & Burley, 1998; Keeley, 2012). Pine bark with thickness >15 mm can protect its cambium for up to 3 min against surface fires when temperatures reach 400°C (He et al., 2012). These pines delay the time gap between building surface fuel load and canopy fire by synchronization of their taller height and self‐pruning of dead branches. Pine seeds germinate better on bare soil after surface fires (Keeley, 2012). The reduced number of repeated fires due to limited availability of crown fire loads facilitates the recruitment of pine seedlings close to parent trees (Keeley, 2012). *Pinus sylvestris* in Europe and Asia, *P. pinea* and *P. pinaster* in Europe, and *P. ponderosa* in North America are examples of taxa with fire‐tolerater strategies (Keeley, 2012). An interesting life‐history trait in some pines is the delayed development (5–10 years) of the trunk; this is known as the “grass stage” (Keeley, 2012). Pines with this strategy accumulate needles over apical buds to protect them against fire (He et al., 2012). A thick tuft of green needles protects the meristem in the seedlings of *P. palustris* in the coastal plains of southern United States and in *P. merkusii* in eastern Himalaya and South‐East Asia. With the appearance of a perennial bunch grass for several years, pine seedlings at the grass stage develop a deep root system. After the release from this stage, seedlings may grow by about 1.5 m in a couple of years, and trees that develop from them may live for hundreds of years (Platt, Evans, & Rathbun, 1988). Such adaptations in pines arose between 126 and 89 Myr (He et al., 2012), indicating either that fire acted as an evolutionary agent much earlier or that these characters already existed in pines and proved advantageous in fire‐prone ecosystems.

The second category of fire‐adapted pines identified by Keeley (2012) is fire‐embracing pines, which employ crown fire to promote the intense fires that consume tree canopies. Retention of dead wood by these pines in their crowns to promote crown fire results in postfire regeneration of serotinous seeds (Keeley, 2012). Serotiny, a pivotal fire‐embracer trait, allows pines to release seeds in large numbers after intense fires (He et al., 2012). Besides pines, serotiny is also exhibited in the family Pinaceae by *Picea mariana* and probably by *Larix gmelinii* (He et al., 2012). However, most pines (85 out of the 115 species) are nonserotinous (Asia, 29 out of 33 species; Europe, 9 out of 13 species; North America, 27 out of 40 species; and Central America, 20 out of 29 species are nonserotinous; McGregor et al., 2012).

Similar to fire‐tolerater or fire‐embracer pines, fire‐refugia pines (e.g., *P. sabiniana* in California, USA) have thick bark and retain dead branches in the crown, but lack cone serotiny that characterizes fire‐embracer pines (Keeley, 2012). Fire‐refugia pines can grow well in grasslands and chaparral, rock outcrops, or riparian areas (Keeley, 2012). These strategies in pines, developed in the Late Cretaceous, enabled them to survive in the fire‐prone productive habitats dominated by angiosperms.

Fire can also have negative impacts on pines and reduce the area occupied by them. *Pinus nigra*, for example, was a dominant species in the northern Iberian Plateau of Spain during the early Holocene. There was, however, a major decline in the extent of forests of this species in 1,300–1,200 cal BP due to human‐associated fires and intense farming (Morales‐Molino, Tinner, Garcia‐Anton, & Colombaroli, 2017).

### 2.2 Pine adaptations for extreme environments

Buds and the bark of many evergreen pines (e.g., *P. aristata, P. contorta, P. koraiensis, P. monticola, P. mugo, P. parviflora, P. peuce, P. pumila* and *P. rostrata*) can survive at −90°C (Strimbeck, Schaberg, Fossdal, Schröder, & Kjellsen, 2015). *Haploxylon* pines can establish in extreme environments because they are well adapted to dry and cold habitats that seldom experience fire (Keeley, 2012). Waring and Franklin (1979) asked what drives the evolution of coniferous‐dominated forests compared to the deciduous hardwood forests in the temperate regions. They found that height (large trees) and the evergreen needles of conifers provide a buffer against nutrient‐ and moisture‐stressed habitats. The longer juvenile period in *Haploxylon* pines (McGregor et al., 2012; Yeaton, 1978) may be the result of a conservative strategy prioritizing individual survival over early reproduction. Also, longer life spans in *Haploxylon* taxa (McGregor et al., 2012) may indicate that they belong in more stressful habitats, as longevity and establishment in stressful environments have been positively correlated (Strauss & Ledig, 1985; Valladares & Niinemets, 2008). Also, larger seeds have been related to higher tolerance to different types of stress, such as drought or shade (Baker, 1972; Grime, 1965; Westoby, Falster, Moles, Vesk, & Wright, 2002; Wright et al., 2010) and *Haploxylon* pines tend to have bigger seeds than *Diploxylon* taxa (McGregor et al., 2012; Tomback & Linhart, 1990; Yeaton, 1978). Pines in stressful conditions face difficulties in locating safe sites for the regeneration of seedlings (Keeley, 2012). In this context, dispersal by animals provides a more effective way of finding suitable regeneration sites (Tomback & Linhart, 1990), and this mode of dispersal is much more common in *Haploxylon* than in *Diploxylon* pines (McGregor et al., 2012; Tomback & Linhart, 1990). For example, the bird *Nucifraga columbiana* efficiently disperses seeds of *P. edulis* in north‐central Arizona (Vander Wall & Balda, 1977) and seeds of *P. albicaulis* in California (Tomback, 1982), and the red squirrel (*Sciurus vulgaris orientis*) disperses seeds of *P. koraiensis* in Japan (Hayashida, 1989). Also, the seeds of *P. cembra* are dispersed by both *N. caryocatactes* and *S. vulgaris* in the Italian Alps (Zong et al., 2010).

### 2.3 Ectomycorrhizal associations

The success of pines in temperate northern latitudes and at higher altitudes in the tropics has been attributed to the replacement of arbuscular mycorrhizal (AM) forests rich in tree diversity with ectomycorrhizal (EM) forests rich in fungal diversity but low in tree diversity, often culminating in vast monodominant stands of Pinaceae (Malloch, Pirozynski, & Raven, 1980). Many traits extant in Pinaceae were common throughout the group until subsequent divergences triggered by environmental conditions (Burleigh & Matthews, 2004; Chaw, Parkinson, Cheng, Vincent, & Palmer, 2000). Ectomycorrhizal associations were common in early gymnosperms, such as members of Gnetales and Cupressaceae. These turned out to be an apomorphic character and were only employed by Pinaceae for supporting establishment and sustenance under conditions of nutrient and water stress. The oldest fossils of EM roots of gymnosperms are from about 50 Myr ago in the Middle Eocene and are associated with *Pinus* (Le Page, Currah, Stockey, & Rothwel, 1997). Development of pine‐ectomycorrhizal associations coincides with diversification, establishment, and dominance of pines progressively at different stages in the history of its evolution. About 270 Myr ago in the Permian, gymnosperms in the families, Gnetaceae or Aurocariaceae, shifted habitat preferences toward mesic sites and their stress tolerance mechanisms became redundant (Gao & Lan, 2016; Wan et al., 2018). Ectomycorrhiza played an important role in the diversification of pines in the Late Cretaceous, particularly in extreme environments.

Pines probably originated in the mid‐Mesozoic Era (Keeley, 2012). Radiations in basidiomycetes during the Paleogene and Neogene (Bruns et al., 1998) gave rise to fungal species in taxa such as *Rhizopogon* and *Suillus* which formed EM associations with the rapidly diverging Pinaceae. The coincidence in the timing of diversification between EM and pine provides support for the notion that mycorrhizal support was a key factor in pine diversification. Palaeotropic‐specific clades of Inocybaceae diversified during the Cretaceous and early Paleogene in response to angiosperm diversification but also showed a transition to association with Pinaceae during the Paleogene (Matheny et al., 2008). Cooler climatic conditions aided in the spread of pines and Fagales. Since both groups are obligately EM, this helped in dispersal and further diversification of the fungi involved in EM (Berggren & Prothero, 1992; Malloch et al., 1980).

## 3 WHY HAVE PINES BECOME THE DOMINANT CONIFER TAXON IN MANY ENVIRONMENTS OVER EVOLUTIONARY TIME?

Pines were able to colonize large areas after the glacial era (Macdonald, Cwynar, & Whitlock, 1998). *Pinus ponderosa* experienced a long delay establishment, followed by rapid expansion across large parts of the central Rocky Mountains in North America, suggesting climate‐ rather than dispersal‐driven expansion (Norris, Betancourt, & Jackson, 2016). Desponts and Payette (1993) reconstructed the postglacial history of *P. banksiana* and found that this species formed small stands from 300 to 2,400 B.P. and that regional expansion of its range occurred between 2,400 and 1,750 B.P. Carrión, Sánchez‐Gómez, Mota, Yii, and Chaín (2003) reconstructed the vegetation history of Sierra de Gádor, southern Spain, and found pollen spectra for the period *ca*. 6,850–6,060 cal yr. B.P. to be dominated by pines; they observed three phases of decline at *ca*. 6,750, 6,550, and 6,350 cal yr. B.P. An increase in pine pollen was observed up to *ca*. 1,700 cal yr. B.P., but pine then declined, with an increase in oaks, Cupressaceae and Poaceae. Pine increased and oak declined during the period *ca*. 3,940–1,760 cal yr. B.P. Fire events played a significant role during this period when increased fire intensity and human activity might have facilitated pines during *ca*. 1,760–1,620 cal yr. B.P. (Carrión et al., 2003).

Pines have expanded their ranges in many parts of the Northern Hemisphere during the Anthropocene. For example, native *P. contorta* has expanded by invading high‐altitude meadows in many parts of the western United States and *P. ponderosa* has established in forest–grassland ecotones in Colorado, USA (Rundel et al., 2014). Pine plantations now cover huge areas outside the native range of the genus in the Southern Hemisphere, where they are planted mostly for timber and pulp. There are about 2 million ha of *P. radiata* plantations in Chile and 1.2 million ha of *P. taeda* in Brazil (Nuñez et al., 2017; Simberloff et al., 2010). Invasive pines are largely *Diploxylon* taxa, belonging to lineages that were good colonizers in the past (Gallien et al., 2016). Climate could be an important determinant for predicting the potential area that could be invaded by pines, but expansion and invasion of pines in introduced ranges are also mediated by seed predation, propagule pressure, mycorrhiza, and competition with resident species in recipient communities (Nuñez & Medley, 2011; Nuñez, Simberloff, & Relva, 2008; Richardson, 2006; Richardson & Bond, 1991; Richardson et al., 1994). Human activities such as altered fire regimes, construction activities, altered land‐use practices, establishment of plantations, manipulation of natural ecosystems, alteration of soil biota through species reshuffling, and anthropogenic pollution exert negative impacts on certain pines by reducing their range and dominance (Richardson et al., 2007). Below, we discuss that in addition to the adaptations to fire and extreme environments that drove pine diversification and spread in the Late Cretaceous, fire characteristics, mycorrhizal associations, ecosystem processes, biogeographical**–**evolutionary advantages, and diverse human activities are now interacting in complex ways to mediate pine dynamics (Figure 3).

### 3.1 Fire as a facilitator of pine expansion and invasion

The extraordinary potential of pines to establish and spread following fire and to alter fire regimes has helped them to dominate globally (Raffaele, Nuñez, Enestron, & Blackhall, 2016). Fire‐adapted traits of pines that helped them to survive and spread in the Late Cretaceous during angiosperm emergence continue to facilitate their spread in the Anthropocene (Figure 3). Flammable long needles of *P. palustris* exert huge impacts by increasing the temperature and extending the long periods of heat, which negatively affect other trees in pine savanna (Ellair & Platt, 2013). The question is whether fire‐adapted pines exploit fire as an ecological driver for their survival and spread (thereby being effectively “passengers of change”) or whether they utilize fire in a novel way to gain dominance (thereby “driving change”).

Fire, a key ecological disturbance, facilitates the spread of pines in their nonnative ranges (Franzese & Raffaele, 2017). Invasive pines can alter spread, severity, flammability and frequency of fire, and fuel loads (Mandle, Bufford, Schmidt, & Daehler, 2011; Paritsis et al., 2018; Taylor et al., 2017). Fire‐adapted invasive pines exert positive feedbacks on fire, and fire in turn can stimulate pine regeneration (Baker, 2009; Richardson & Cowling, 1992). Although some communities are resilient to invasion of pines followed by fire (Nuñez & Raffaele, 2007), other communities can be replaced with fire‐adapted communities as a consequence of the invasion of fire‐adapted pines. Taylor et al. (2017) studied the positive feedbacks between fire and *P. contorta* invasion in Argentina, Chile, and New Zealand. Dense *P. contorta* stands have massive fire loads which result in severe fires and intense soil heating which favors *P. contorta* regeneration but hinders regeneration of fire‐sensitive native Patagonian trees. Severe fires in *P. contorta* stands eliminate herbaceous cover, thereby reducing competition which favors *P. contorta* whose seedlings are susceptible to competition with grasses (Taylor et al., 2017). Positive fire–pine feedbacks help to understand the role of fire in the invasion of pines in the Southern Hemisphere.

Fire may also be a driver of range contraction of certain pines. *Pinus nigra* is reported to be sensitive to intense fires and lacks traits such as serotiny; this allows the expansion of oak woodlands into forests once dominated by *P. nigra* (Morales‐Molino et al., 2017). *Pinus palustris* was historically dominant on the coastal plains of the southeastern United States (Chandler, 2014), but currently covers only about 2% of its original range (Means, 1996). Human‐assisted fire may play a key role in degrading *Haploxylon* pine forests (Chandler, 2014). Stralberg et al. (2018) predicted that most of Alberta's natural regions in Canada are likely to be converted into deciduous woodlands and grasslands within a century. They argued that rising summer temperatures and reduced soil moisture, which lengthens the wildfire season, are key drivers of vegetation change in the boreal forests of Alberta.

### 3.2 Life‐history and functional traits

Pines often form monocultures, both in their native and nonnative ranges, and exert varied impacts in invaded ecosystems such as dramatic alterations to fire and hydrological regimes, changed soil nutrients, and aboveground and belowground communities (Nuñez et al., 2017; Simberloff et al., 2010). The causes of species monodominance vary between habitats. Mycorrhizal associations play an important role in the species monodominance in tropical forests (Corrales, Mangan, Tuner, & Dalling, 2016; Peh, Lewis, & Lloyd, 2011). In this section, we discuss briefly the impact of adaptation/traits in pines, ecosystem process, mutualistic associations, and biogeographical advantages in the current rising dominance of pines in many parts of the world.

Pines have various physiological traits and adaptations that have allowed them to survive in extreme environments, for example, under drought conditions. The ability of pines to close stomata at relatively higher (less negative) water potential enables them to survive in dry habitats (Singh, Zobel, Garkoti, Tewari, & Negi, 2006). Since pine seeds are desiccation‐tolerant, their regeneration is less affected by drought. Pines also need not use evaporative cooling to regulate leaf temperatures; they can afford to close their stomata on hot and dry days (Waring & Schlesinger, 1985). Native fire‐intolerant oaks (e.g., *Quercus laevis*) facilitate the establishment of newly germinated seedlings of fire‐adapted *P. palustris* in the xeric sites of southeastern United States (Loudermilk et al., 2016). Adults of *P. palustris* are not sensitive to drought because they are capable of hydrolytic lift but adult pines have high fire loads which results in severe fire which is often detrimental to young pine seedlings (Espeleta et al., 2004; Grace & Platt, 1995; Taylor et al., 2017). Litter build‐up by *P. palustris* helps to retain water and improve nutrient availability (Harrington, 2006). Although *P. palustris* has a grass stage that lasts up to 20 years, its 1‐ to 2‐year‐old seedlings are sensitive to drought (Loudermilk et al., 2016). The establishment and early growth of *P. palustris* seem to be facilitated by oaks (Loudermilk et al., 2016). *Quercus laevis,* a native midstorey oak and often associated with *P. palustris,* can facilitate pine seedling growth and survival by hydrolytic lift, thereby acting as a nurse plant (Loudermilk et al., 2016). Species such as *Q. rubra,* which occurs in mixed forests with *P. banksiana*, is fire‐tolerant and is better adapted to xeric conditions than pines due to root sprouting. Pines, however, outcompete oaks due to their vigorous postfire recruitment and eventually attain greater heights and thus overshadow the shorter statured oak (Frelich, Reich, & Peterson, 2017). A better understanding of positive interactions between fire‐intolerant natives and fire‐tolerant pines or between fire and pines is needed to design effective management strategies.

Expansion and invasion of pines may be limited until the onset of favorable conditions. A time lag in the invasion of pines following fire could be due to lack of mycorrhizae or absence of vegetation (Nuñez et al., 2009, 2013; Raffaele et al., 2016; Richardson et al., 1994). Dovčiak, Frelich, and Reich (2005) studied the two‐phased invasion of *P. strobus* (white pine) into drought‐prone and nutrient‐poor old fields with oak savanna in the north‐central United States. The first phase of *P. strobus* expansion occurred during a period of favorable climate, which allowed pines to establish in shaded forest edges. A second phase occurred about 5 years later when high precipitation and cooler conditions facilitated the spread of *P. strobus* into open fields devoid of trees. These authors identified three successional pathways in *P. strobus* expansion, first, slow and creeping spreading of *P. strobus* with low seed rain into shaded forest edges, and second, abundant seed rain which facilitates rapid *P. strobus* expansion. In the third pathway, no or very little expansion occurs due to low seed rain and lack of shade conditions, which allows grasses to establish. This study illustrates the importance of seed rain, shade, and climate in *P. strobus* colonization. Invasion of *P. contorta* in Chilean Patagonia resulted in the selection of shade‐tolerant species with conservation of reproductive traits such as heavier seeds, epizoochorous seed dispersal, higher plant height, and different fruit types (Bravo‐Monasterio, Pauchard, & Fajardo, 2016).

#### 3.2.1 Ectomycorrhizal associations

Ectomycorrhizal associations are important mediators of pine establishment and spread in native and nonnative ranges (Nuñez et al., 2009). EM trees experience positive feedbacks under canopies of their conspecifics, but AM trees experience negative feedbacks in soil beneath conspecifics (Bennett et al., 2017). Positive feedbacks to EM trees could be due to the ability of EM to channel nitrogen to their host in nitrogen‐poor soils compared to AM (Corrales et al., 2016). Ectomycorrhizal fungi produce N‐degrading enzymes that give them greater access to organic N compared to AM fungi (Nashölm et al., 1998; Read & Perez‐Moreno, 2003), thus enabling pines to access organic N. This difference between AM and EM trees allows pines to become dominant species with great ecosystems impacts.

*Pinus contorta* has been introduced to many parts of the world. Nonnative mammals present in some parts of the introduced range of *P. contorta* have helped to spread Northern Hemisphere ectomycorrhizal fungi that were cointroduced with the pine, thereby facilitating invasion (Nuñez et al., 2013; Wood et al., 2015). In this case, EM spread is independent of pines but the dispersal of EM by nonnative mammals helps pines to expand their ranges. Native trees, however, did not develop associations with nonnative EM, which are associated with *P. contorta*. Therefore, only the pines can use their own mycorrhizal fungi, which excludes native species. This supports facilitation in pines, in accordance with the invasional meltdown hypothesis (Simberloff & von Holle, 1999).

#### 3.2.2 Ecosystem processes

Litterfall influences ecosystem processes such as nutrient cycling, which mediates the range expansion of pines in their native range (Read & Perez‐Moreno, 2003). Litter and canopies of *P. ponderosa* have a negative impact on the growth and establishment of the invasive shrub species *Centaurea stoebe* by modifying soil and nutrient availability and its allelopathic effects (Metlen & Callaway, 2015). Negative impacts of *P. contorta* on native Chilean Patagonia vegetation are determined by its height and canopy size (Franzese, Urrutia, García, Taylor, & Pauchard, 2017).

Pines invade treeless temperate grasslands and fynbos shrublands (Figure 2g,h) and have significant impacts on nutrient cycling, carbon sequestration, and ecohydrology (Rundel et al., 2014). Gymnosperms dominate in low‐nutrient situations and angiosperm dominance in the productive habitats (Berendse & Scheffer, 2009). The low nutrient requirements of pines, and their ability to mobilize soil nutrients, allow them to outcompete broad‐leaved species with higher nutrient demands. In central Himalaya, the net primary productivity per unit foliar nitrogen in *P. roxburghii* forest is 2.3–4.5 times more than those of oak (*Q. leucotrichophora*), śāl (*Shorea robusta*), and other forest types (Singh & Singh, 1992). *Pinus strobus* invades nitrogen‐limited grasslands by having a longer nitrogen residence time which keeps the annual demand lower than for all other species including other tree species and grasses (Laungani & Knops, 2009). Thus, nitrogen retention is probably one of the drivers of range expansion of pines. Keeping nutrient availability low is one of the several strategies where conifers have an advantage over more nutrient‐demanding plants in fertile sites (Berendse & Scheffer, 2009).

The infertile and acidic soils from the oldest coastal terraces in northern California support pygmy conifers (e.g., *P. contorta* var. *bolanderi*,* P. muricata,* and *Cupressus pygmaea*) and certain ericaceous species (Northup, Dahlgren, Aide, & Zimmerman, 1999). Northup, Yu, Dahlgren, and Vogt (1995) found that *P. muricata* releases polyphenols in the infertile soils of heath forests which helps in the release of dissolved organic nitrogen rather than ${\text{NO}}_{3}^{-}$/${\text{NH}}_{4}^{+}$. *P. muricata* thus survives in the extremely harsh conditions by using a nitrogen conservation mechanism in an ecosystem with severe N deficiency. Pines can utilize organic nitrogen through their mycorrhizal symbionts. Pines seem to conserve nitrogen in infertile soils by producing polyphenol (tannin)‐rich litter (Hättenschwiler & Vitousek, 2000). The capacity of tannin to precipitate protein, however, is mediated by the composition, hydroxylation, substitution, polymerization, and linkage connecting monomer units of condensed tannins (Suseela & Tharayil, 2017). It is not clear why conifers are abundant in old terraces worldwide (Coomes et al., 2005). More evidence is required to clarify whether condensed tannin‐driven short‐circuiting of the nitrogen cycle by pines gives them advantages over their competitors.

#### 3.2.3 Biogeographical–evolutionary advantages

Many of the hypotheses that are currently debated in invasion ecology assume that species experience biogeographical**–**evolutionary advantages in their introduced ranges compared to their native ranges (Hierro, Maron, & Callaway, 2005; Inderjit, Catford, Kalisz, Simberloff, & Wardle, 2017; Inderjit, Wardle, Karban, & Callaway, 2011). Gallien et al. (2016) reported that understanding biogeographical and evolutionary histories is valuable for understanding pine invasions. They found that pines belonging to lineages that were particularly successful at colonizing new regions over the evolutionary history of the genus are more likely to be invasive. The role of climate‐niche evolution in pine invasion has also been clearly demonstrated. Gallien et al. (2016) argued that an expansion of pines in the climatic niche between native and invasive ranges may be driven not by local adaptation, but by the potential of pines to spread in climatic conditions that are not available in native ranges. Pines may experience these advantages in terms of their responses to consumers, competitors, or mutualists in novel ranges (Taylor et al., 2017; Wood et al., 2015). Pines suppress species richness in both native and introduced ranges but can grow much faster in introduced ranges than native ranges (Taylor et al., 2016b). *Pinus contorta* exerts greater impact on species richness of individual species and impact on composition of native species along invasion gradient in introduced ranges than in the native range (Taylor et al., 2016b). We have limited empirical evidence to identify the mechanisms behind advantages experienced by pines in their introduced ranges.

Pines may not always experience enemy release (*sensu* Keane & Crawley, 2002) in their introduced ranges. *Pinus radiata* was introduced to Spain where it currently coexists with the native *P. pinaster* (Moreira, Zas, & Sampedro, 2013). Zas, Moreira, and Sampedro (2011) found that the pine weevil *Hylobius abietis* in the coastal area of NW Spain prefers the native *P. pinaster* to the introduced *P. radiata*. Greater damage to the nonnative pine, despite the preference of the weevil for the native pine, was attributed to the lack of inducible defense due to effect of genetic bottleneck in the nonnative species. Moreira et al. (2013), however, found that native herbivores supported by *P. pinaster* use *P. radiata* as a host; this suggests genetic constraints on the evolution of resistance against herbivores in its introduced range.

More research is needed on biogeographical**–**evolutionary advantages experienced by nonnative pine species in their introduced ranges. Pine invasions occur predominantly in treeless ecosystems where the lack of native trees reduces competition for the nonnative pines (Rundel et al., 2014). In Argentina and Chile, there are several cases of invasion in the Patagonian steppe, an arid environment dominated by grasses and shrubs where no native trees grow (Langdon et al., 2010; Sarasola et al., 2006). South African fynbos, another virtually treeless ecosystem, is highly invaded by pines (Richardson & Brown, 1986; Richardson, Cowling, & Le Maitre, 1990). In New Zealand, large areas of grasslands and shrublands have been invaded by *P. contorta* (Ledgard, 2001). Richardson et al. (1994) reviewed the habitats invaded by the different pine species in the Southern Hemisphere; they found that the absence of native trees, due to natural factors or human activities, is a very common characteristic of invaded habitats. In alpine ecosystems, pines may also have an advantage if they can grow above the native tree line. In Chile and New Zealand, native trees can grow up to 1,950 m.a.s.l. and 1,350 m.a.s.l., respectively, while pines can grow up to 4,000 m.a.s.l. in their native range (Körner & Paulsen, 2004). This capability of pines to endure the stressful conditions of alpine environments may help them invade above tree line in their introduced range. Invasions above tree line have already occurred along the Andean range (Pauchard et al., 2015) and in New Zealand (Simberloff et al., 2010). The genus *Pinus* is an excellent model system for designing experiments to unravel the mechanisms of range expansion in native range and invasion in introduced ranges (Richardson, 2006). More data are needed to determine whether pines experience plant–soil feedbacks, enemy release, novel chemicals and litter decomposition, and nutrient availability in their introduced ranges.

Pines may resist the invasion of nonnative species in their native ranges. *Pinus ponderosa* exhibits strong competitive potential in its native range in North America and resists invasion by some aggressive nonnative species. Acidic conditions and low phosphorus levels under canopies of native *P. ponderosa* in the northern Rocky Mountains favor native grasses such as *Festuca idahoensis* and *Pseudoroengaeria spicata* (Gundale, Sutherland, & DeLuca, 2008), and the aggressive Eurasian species *Centaurea stoebe* does not invade in the habitat where *P. ponderosa* was present (Metlen & Callaway, 2015). Pine litter is known to exhibit allelopathic potential (Lodhi & Killingbeck, 1982), and this could be the mechanism for preventing the establishment of *C. stoebe* (Metlen & Callaway, 2015). This supports a key invasion hypothesis: “biotic resistance to invasion.” Biotic resistance to invasion by soil microbial communities is widely studied (see Inderjit & van der Putten, 2010), but few studies have explored the role of chemicals produced by native species in resisting invasion by other species. More research is needed to establish the role of chemicals released by pines in resisting invasion by nonnative plant species.

### 3.3 Human‐mediated pine dominance

Pines have flourished in many areas during the Anthropocene due to many factors associated with the accelerating impacts of humans on ecosystems. Increased fire intensity, human‐mediated disturbance, and climate change have dramatically altered opportunities for pine establishment and spread (Carrión et al., 2003). *Pinus* is the most widely planted tree genus in the world (Brown & Ball, 2000); this has provided propagule pressure to launch invasions in many areas (Braga, Zenni, & Hay, 2014; Essl, Moser, Dullinger, Mang, & Hulme, 2010; Nuñez et al., 2009, 2017; Pauchard et al., 2016; Procheş et al., 2012; Simberloff et al., 2010). Such human‐mediated movements and the capacity of pines to change nutrient cycling and other functional traits have enabled them to persist, expand, and dominate in many environments in the Northern and Southern Hemispheres, and pines are widely invasive in the Southern Hemisphere.

Pines owe their invasiveness to small seed mass, propagule pressure, rapid population growth (Gallien et al., 2016; Rejmánek & Richardson, 1996), wide niche breadth (McGregor et al., 2012), and widespread and sustained human use (Procheş et al., 2012). Some pine taxa (e.g., *P. contorta*,* P. densiflora*,* P. halepensis*,* P. pinaster*, and *P. radiata*) are light‐demanding and fast‐growing; these species regenerate abundantly as even‐aged cohorts following natural or human‐mediated disturbances and differ from other conifers in their ability to aggressively colonize disturbed sites (Richardson, 1998). Although many facets of the invasion ecology of pines have been studied in many habitats around the world, more work is needed to expand our knowledge of the full range of factors that mediate success. For example, further research is needed to determine the roles of pine chemicals, litter‐manipulated ecosystem processes (plant–soil feedbacks), and biogeographical**–**evolutionary advantages gained by pines in their introduced ranges.

One element of human‐mediated global change, global warming, could increase forest fire by increasing drought frequency and could cause earlier onset of the growing season (Westerling, Hidalgo, Cayan, & Swetnam, 2006). Scots pine (*P. sylvestris*) was dominant in the early Holocene when temperatures were 2.5°C warmer than in late 19th century (Kullman & Kjällgren, 2006). Pines are likely to regain dominance in tree line ecotones in response to changing climate regimes, as occurred in the early Holocene (Kullman & Kjällgren, 2006). Pines may shift or expand their ranges or regain dominance in response to climate change.

Human impacts such as alterations to fire and grazing regimes and land use, plantations, the reshuffling of biotas, and pollution could reduce or shift pine ranges (Richardson et al., 2007). Grazing by rats, rabbits, sheep, and cattle exerts negative impacts on the regeneration of pine seedlings in many areas (e.g., *P. contorta*,* P. radiata* var*. binata,* and *P. sylvestris*) (Nasca, Relva, & Núñez, 2018; Richardson et al., 2007). Several pine taxa with small ranges are facing the threat of extinction through habitat transformation, heavy utilization, and other factors associated with human actions. Richardson et al. (2007) discussed examples of factors that can result in the reduction and/or shift in pine ranges.

## 4 CONCLUSIONS

Adaptations to fire regimes in the Late Cretaceous mediated the diversification of pines, and fire as an ecological disturbance drives the current pine expansion. Traits that evolved in response to angiosperm‐fuelled fire cycles continue to drive pine expansion in the Anthropocene. Adaptations to fire such as serotiny, self‐pruning of dead branches, long needles, and the grass stage helped pine to survive and flourish in fire‐prone environments in the Late Cretaceous (Figure 3). Pines developed traits and diversified in extreme environments where angiosperms could not survive. These traits include the following: adaptation to premonsoon drought, the capacity to close stomata on hot and dry days and at high water potentials, various ruderal strategies, vigorous regeneration, high competitive abilities, and reduced growth and acclimatization during drought (Figure 3). Traits such as enormous seed pressure, height, fast‐growing, ectomycorrhiza, or thick bark contribute to the survival and dominance of pines. Intentional and accidental fires related to practices in agriculture, forestry, and human settlements have the potential to trigger or enhance pine invasion (Le Page, Oom, Silva, Jönsson, & Pereira, 2010). Land‐use change is providing conditions that allow for the successful implementations of fire strategies that have been developed in course of the evolution of *Diploxylon* pines. The transition from hardwood forests to habitats dominated by pines was aided by shifting cultivation and land abandonment (Delcourt, Delcourt, Ison, Sharp, & Gremillion, 1998). With man‐made fires now more important than wildfires in many parts of the current range of pines (e.g., 84% of fire events and 44% of the total burnt area in the United States; Balch et al., 2017), fire‐adapted species are being favored. Century‐old traditions relating to land usage and characteristic vegetation of North American tallgrass prairies have seen increase in fire frequencies, leading to the exclusion of fire‐sensitive species (Grimm, 1984). In ecosystems with conditions suitable for wildfires, litter quality and quantity limit such incidents. The introduction of species associated with flammable fuel load can alter fire regimes and lead to vegetation change (Thonicke, Venevsky, Sitch, & Cramer, 2001).

Ecological, evolutionary, and diverse human‐mediated factors have interacted to mediate the range of pines over evolutionary time and in recent centuries with increased influence of humans (Figure 3). Pines have a complicated ecology that combines two principal strategies: (a) adaptations to various fire regimes and the effect of fire as an ecological disturbance; and (b) adaptations to extreme conditions. The management of pine‐dominated ecosystems in both the native and adventive range of the genus is facing increasing challenges in the Anthropocene (Brundu & Richardson, 2016; Nuñez et al., 2017; Richardson, 1998; Richardson et al., 2007). Insights from all the processes shown in Figure 3 are needed to formulate sustainable strategies for management to meet diverse goals relating to biodiversity and ecosystem functioning.

## CONFLICT OF INTEREST

None declared.

## AUTHORS’ CONTRIBUTIONS

SPS initiated the idea for this review. Inderjit prepared the first draft. All authors provided inputs and commented on subsequent versions. SM prepared Figure 1, DMR compiled Figure 2 and Inderjit produced Figure 3.

## DATA ACCESSIBILITY

This is a review article, and we have not included any data in a publicly accessible repository.

## References

[ece34499-bib-0001] Améztegui, A. , Brotons, L. , & Coll, L. (2010). Land‐use changes as major drivers of mountain pine (*Pinus uncinata* Ram.) expansion in the Pyrenees. Global Ecology and Biogeography, 19, 632–641. 10.1111/j.1466-8238.2010.00550.x

[ece34499-bib-0002] Augusto, L. , Davies, T. J. , Delzon, S. , & De Schrijver, A. (2014). The enigma of the rise of angiosperms: Can we untie the knots? Ecology Letters, 17, 1326–1338. 10.1111/ele.12323 24975818

[ece34499-bib-0003] Averill, C. , Turner, B. L. , & Finzi, A. C. (2014). Mycorrhiza‐mediated plants and decomposers derives soil carbon storage. Nature, 505, 543–546. 10.1038/nature12901 24402225

[ece34499-bib-0201] Averill, C. , Turner, B. L. , & Finzi, A. C. (2014). Mycorrhiza‐mediated competition between plants and decomposers drives soil carbon storage. Nature, 505, 543–545. 10.1038/nature12901 24402225

[ece34499-bib-0004] Baker, H. G. (1972). Seed weight in relation to environmental conditions in California. Ecology, 53, 997–1010. 10.2307/1935413

[ece34499-bib-0005] Baker, W. L. (2009). Fire ecology in Rocky Mountain landscapes. Washington, DC: Island Press.

[ece34499-bib-0006] Balch, J. K. , Bradley, B. A. , Abatzoglou, J. T. , Nagy, R. C. , Fusco, E. J. , & Mahood, A. L. (2017). Human‐started wildfires expand fire niches across the United States. Proceedings of the National Academy of Sciences of the United States of America, 114, 2946–2951. 10.1073/pnas.1617394114 28242690 PMC5358354

[ece34499-bib-0007] Bennett, J. A. , Maherali, H. , Reinhart, K. O. , Lakberg, Y. , Hart, M. M. , & Klironomos, J. (2017). Plant‐soil feedbacks and mycorrhizal type influence temperate forest population dynamics. Science, 355, 181–184. 10.1126/science.aai8212 28082590

[ece34499-bib-0008] Berendse, F. , & Scheffer, M. (2009). The angiosperm radiation revisited, an ecological explanation for Darwin's ‘abominable mystery’. Ecology Letters, 12, 865–872. 10.1111/j.1461-0248.2009.01342.x 19572916 PMC2777257

[ece34499-bib-0009] Berggren, W. A. , & Prothero, D. R. (1992). Eocene‐Oligocene climatic and biotic evolution. Princeton, NJ: Princeton University Press.

[ece34499-bib-0010] Bond, W. J. , & Scott, A. C. (2010). Fire and the spread of flowering plants in the Cretaceous. New Phytologist, 188, 1137–1150. 10.1111/j.1469-8137.2010.03418.x 20819174

[ece34499-bib-0011] Braga, E. P. , Zenni, R. D. , & Hay, J. D. (2014). A new invasion species in Southern America: *Pinus oocarpa* Schiede ex Schtdl. BioInvasions Records, 3, 207–211. 10.3391/bir.2014.3.3.12

[ece34499-bib-0012] Bravo‐Monasterio, P. , Pauchard, A. , & Fajardo, A. (2016). *Pinus contorta* invasion into treeless steppe reduces species richness and alters species traits of the local community. Biological Invasions, 18, 1883–1894. 10.1007/s10530-016-1131-4

[ece34499-bib-0014] Brodribb, T. , Pittermann, J. , & Coomes, D. A. (2012). Elegance versus speed: Examining the competition between conifer and angiosperm trees. International Journal of Plant Sciences, 173, 673–694.

[ece34499-bib-0202] Brown, C. , & Ball, J . (2000). World view of plantation grown wood In Krishnapillay B., Soepadmo E., Arshad N.L., Wong A., Appanah S., Chik S.E., Manokaran N., Tong H.L. & Choon K.K. (Eds.). Forests and society: the role of research (pp. 377–389). XXI IUFRO world congress, August 2000, Kuala Lumpur, Malaysia, Vol 1. Vienna and Kuala Lumpur: IUFRO and FRIM.

[ece34499-bib-0015] Brundu, G. , & Richardson, D. M. (2016). Planted forests and invasive alien trees in Europe: A Code for managing existing and future plantings to mitigate the risk of negative impacts from invasions. Neobiota, 30, 5–47. 10.3897/neobiota.30.7015

[ece34499-bib-0016] Bruns, T. D. , Szaro, T. M. , Gardes, M. , Cullings, K. W. , Pan, J. J. , Taylor, D. L. , … Lişş, Y. (1998). A sequence database for the identification of ectomycorrhizal basidiomycetes by phylogenetic analysis. Molecular Ecology, 7, 257–272. 10.1046/j.1365-294X.1998.00337.x

[ece34499-bib-0017] Burleigh, J. G. , & Matthews, S. (2004). Phylogenetic signal in nucleotide data from seed plants: Implications for resolving the seed plant tree of life. American Journal of Botany, 91, 1599–1613. 10.3732/ajb.91.10.1599 21652311

[ece34499-bib-0018] Carrión, J. S. , Sánchez‐Gómez, P. , Mota, J. F. , Yii, R. , & Chaín, C. (2003). Holocene vegetation dynamics, fire and grazing in the Sierra de Gádor, southern Spain. The Holocene, 13, 839–849.

[ece34499-bib-0019] Certini, G. , & Scalenghe, R. (2015). Holocene as Anthropocene. Science, 349, 246. 10.1126/science.349.6245.246a 26185234

[ece34499-bib-0020] Chandler, H. C. (2014). The effects of climate change and long‐term fire suppression on ephemeral pond communities in southeastern United States. M.Sc Dissertation, Virginia Polytechnic & State University, Blacksburg, VA.

[ece34499-bib-0021] Chaw, S. M. , Parkinson, C. L. , Cheng, Y. , Vincent, T. M. , & Palmer, J. D. (2000). Seed plant phylogeny inferred from all three plant genomes: Monophyly of extant gymnosperms and origin of Gnetales from conifers. Proceedings of the National Academy of Sciences of the United States of America, 97, 4086–4091. 10.1073/pnas.97.8.4086 10760277 PMC18157

[ece34499-bib-0022] Coomes, D. A. , Allen, R. B. , Bentley, W. A. , Burrows, L. E. , Canham, C. D. , Fagan, L. , … Wright, E. F. (2005). The hare, the tortoise and the crocodile: The ecology of angiosperm dominance, conifer persistence and the fern filtering. Journal of Ecology, 93, 918–935. 10.1111/j.1365-2745.2005.01012.x

[ece34499-bib-0023] Corrales, A. , Mangan, S. A. , Tuner, B. L. , & Dalling, J. W. (2016). An ectomycorrhizal nitrogen economy facilitates monodominance in a neotropical forest. Ecology Letters, 19, 383–392. 10.1111/ele.12570 26833573

[ece34499-bib-0024] Delcourt, P. A. , Delcourt, H. R. , Ison, C. R. , Sharp, W. E. , & Gremillion, K. J. (1998). Prehistoric human use of fire, the Eastern Agricultural Complex, and Appalachian oak‐chestnut forests: Paleoecology of Cliff Palace Pond, Kentucky. American Antiquity, 63, 263–278.

[ece34499-bib-0025] Desponts, M. , & Payette, S. (1993). The Holocene dynamics of jack pine at its northern range limit in Quebec. Journal of Ecology, 81, 719–727.

[ece34499-bib-0026] Dovčiak, M. , Frelich, L. E. , & Reich, P. B. (2005). Pathways in old‐field succession to white pine: Seed rain, shade, and climate effects. Ecological Monograph, 75, 363–378. 10.1890/03-0802

[ece34499-bib-0027] Eckert, A. J. , & Hall, B. D. (2006). Phylogeny, historical biogeography, and patterns of diversification for *Pinus* (Pinaceae): Phylogenetic tests of fossil‐based hypotheses. Molecular Phylogenetics and Evolution, 40, 166–182. 10.1016/j.ympev.2006.03.009 16621612

[ece34499-bib-0028] Ellair, D. P. , & Platt, W. J. (2013). Fuel composition influences fire characteristics and understory hardwoods in pine savanna. Journal of Ecology, 101, 192–201. 10.1111/1365-2745.12008

[ece34499-bib-0029] Engelmark, O. , Sjöberg, K. , Andersson, B. , Rosvall, O. , Ågren, G. I. , Baker, W. L. , Barklund, P. , Björkman, C. , Despain, D. G. , Elfving, B. , … Sykes, M. T. (2001). Ecological effects and management aspects of an exotic tree species: The case of lodgepole pine in Sweden. Forest Ecology and Management, 141, 3–13. 10.1016/S0378-1127(00)00498-9

[ece34499-bib-0203] Espeleta, J. , West, J. , & Donovan, L. (2004). Species‐specific patterns of hydraulic lift in co‐occurring adult trees and grasses in a sandhill community. Oecologia, 138, 341–349. 10.1007/s00442-003-1460-8 14689298

[ece34499-bib-0030] Essl, F. , Mang, T. , Dullinger, S. , Moser, D. , & Hulme, P. E. (2011). Macroecological drivers of alien conifer naturalizations worldwide. Ecography, 34, 1076–1084. 10.1111/j.1600-0587.2011.06943.x

[ece34499-bib-0031] Essl, F. , Moser, D. , Dullinger, S. , Mang, T. , & Hulme, P. E. (2010). Selection for commercial forestry determines global patterns of alien conifer invasions. Diversity and Distributions, 16, 911–921. 10.1111/j.1472-4642.2010.00705.x

[ece34499-bib-0032] Fonda, R. W. , Belanger, L. A. , & Burley, L. L. (1998). Burning characteristics of western conifer needles. Northwest Science, 72, 1–9.

[ece34499-bib-0033] Franzese, J. , & Raffaele, E. (2017). Fire as a driver of pine invasions in the Southern Hemisphere: A review. Biological Invasions, 19, 2237–2246. 10.1007/s10530-017-1435-z

[ece34499-bib-0034] Franzese, J. , Urrutia, J. , García, R. A. , Taylor, K. , & Pauchard, A. (2017). Pine invasion impacts on plant diversity in Patagonia: Invader size and invaded habitat matter. Biological Invasions, 19, 1015–1027. 10.1007/s10530-016-1344-6

[ece34499-bib-0035] Frelich, L. E. , Reich, P. B. , & Peterson, D. W. (2017). The changing role of fire in mediating the relationships among oaks, grasslands, mesic temperate forests, and boreal forests in the Lake States. Journal of Sustainable Forestry, 36, 421–432. 10.1080/10549811.2017.1296777

[ece34499-bib-0036] Gallien, L. , Saladin, B. , Boucher, F. C. , Richardson, D. M. , & Zimmermann, N. (2016). Does the legacy of historical biogeography shape current invasiveness in pines? New Phytologist, 209, 1096–1105. 10.1111/nph.13700 26477339

[ece34499-bib-0037] Gao, J. , & Lan, T. (2016). Functional characterization of the late embryogenesis abundant (LEA) protein gene family from *Pinus tabuliformis* (Pinaceae) in *Escherichia coli* . Scientific Reports, 6, 19467 10.1038/srep19467 26781930 PMC4726009

[ece34499-bib-0204] Grace, S.L. , & Platt, W. J. (1995). Neighborhood effects on juveniles in an old‐growth stand of longleaf pine, Pinus palustris. Oikos, 72, 99–105. https://www.jstor.org/stable/3546043

[ece34499-bib-0038] Grime, J. P. (1965). Shade tolerance in flowering plants. Nature, 208, 161–163. 10.1038/208161a0

[ece34499-bib-0039] Grimm, E. C. (1984). Fire and other factors controlling the big woods vegetation of Minnesota in the mid‐nineteenth century. Ecological Monographs, 54, 291–311. 10.2307/1942499

[ece34499-bib-0205] Grotkopp, E. , Rejmánek, M. , & Rost, T. L. (2002). Toward a causal explanation of plant invasiveness: seedling growth and life‐history strategies of 29 pine (Pinus) species. American Naturalist, 159, 396–419. 10.1086/338995 18707424

[ece34499-bib-0040] Gundale, M. J. , Sutherland, S. , & DeLuca, T. H. (2008). Fire, native species, and soil resource interactions regulate the spatio‐temporal invasion pattern of *Bromus tectorum* . Ecography, 31, 201–210. 10.1111/j.0906-7590.2008.5303.x

[ece34499-bib-0041] Harland, M. , Francis, J. E. , Brentnall, S. J. , & Beerling, D. J. (2007). Cretaceous (Albian – Aptian) conifer wood from Northern Hemisphere high latitudes: Forest composition and palaeoclimate. Review of Palaeobotany and Palynology, 143, 167–196. 10.1016/j.revpalbo.2006.07.005

[ece34499-bib-0206] Harrington, T. B. (2006). Plant competition, facilitation, and other overstory‐understory interactions in longleaf pine ecosystems In Jose S., Jokela E. J., & Miller D. L. (Eds.), The Longleaf Pine Ecosystem Ecology, Silviculture, and Restoration (pp. 135–156). New York: Springer.

[ece34499-bib-0042] Hättenschwiler, K. A. , & Vitousek, P. M. (2000). The role of polyphenols in terrestrial ecosystems nutrient cycling. Trends in Ecology & Evolution, 15, 239–243. 10.1016/S0169-5347(00)01861-9 10802549

[ece34499-bib-0043] Hayashida, M. (1989). Seed dispersal by red squirrels and subsequent establishment of Korean pine. Forest Ecology and Management, 28, 115–129. 10.1016/0378-1127(89)90064-9

[ece34499-bib-0044] He, T. , Pausas, J. G. , Belcher, C. M. , Schwilk, D. W. , & Lamont, B. B. (2012). Fire‐adapted traits of *Pinus* arose in the fiery Cretaceous. New Phytologist, 194, 751–759. 10.1111/j.1469-8137.2012.04079.x 22348443

[ece34499-bib-0045] Hierro, J. L. , Maron, J. L. , & Callaway, R. M. (2005). A biogeographical approach to plant invasions: The importance of studying exotics in their introduced and native range. Journal of Ecology, 93, 5–15. 10.1111/j.0022-0477.2004.00953.x

[ece34499-bib-0046] Inderjit , Catford, J. A. , Kalisz, S. , Simberloff, D. , & Wardle, D. A. (2017). A framework for understanding human‐driven vegetation change. Oikos, 126, 1687–1698. 10.1111/oik.04587

[ece34499-bib-0207] Inderjit , & van der Putten, W.H. (2010). Impacts of soil microbial communities on exotic plant invasion. Trends in Ecology & Evolution, 25, 512–519. 10.1016/j.tree.2010.06.006 20638747

[ece34499-bib-0047] Inderjit , Wardle, D. A. , Karban, R. , & Callaway, R. M. (2011). The ecosystem and evolutionary contexts of allelopathy. Trends in Ecology & Evolution, 26, 655–662. 10.1016/j.tree.2011.08.003 21920626

[ece34499-bib-0048] Jakubos, B. , & Romme, W. H. (1993). Invasion of subalpine meadows by lodgepole pine in Yellowstone National Park, Wyoming, U.S.A. Arctic and Alpine Research, 25, 382–390.

[ece34499-bib-0208] Keane, R. M. , & Crawley, M. J. (2002). Exotic plant invasions and the enemy release hypothesis. Trends in Ecology & Evolution, 17, 164–170. 10.1016/S0169-5347(02)02499-0

[ece34499-bib-0050] Keeley, J. E. (2012). Ecology and evolution of pine life histories. Annals of Forestry Science, 69, 445–453. 10.1007/s13595-012-0201-8

[ece34499-bib-0052] Körner, C. , & Paulsen, J. (2004). A world‐wide study of high altitude treeline temperatures. Journal of Biogeography, 31, 713–732. 10.1111/j.1365-2699.2003.01043.x

[ece34499-bib-0053] Kullman, L. , & Kjällgren, L. (2006). Holocene pine tree‐line evolution in the Swedish Scandes: Recent tree‐line rise and climate change in a long‐term perspective. Boreas, 35, 159–168. 10.1111/j.1502-3885.2006.tb01119.x

[ece34499-bib-0054] Langdon, B. , Pauchard, A. , & Aguayo, M. (2010). *Pinus contorta* invasion in the Chilean Patagonia: Local patterns in a global context. Biological Invasions, 12, 3961–3971. 10.1007/s10530-010-9817-5

[ece34499-bib-0055] Laungani, R. , & Knops, J. M. H. (2009). Species‐driven changes in nitrogen cycling can provide a mechanism for plant invasions. Proceedings of the National Academy of Sciences of the United States of America, 106, 12400–12405. 10.1073/pnas.0900921106 19592506 PMC2718360

[ece34499-bib-0056] Le Page, B. A. , Currah, R. S. , Stockey, R. A. , & Rothwel, G. W. (1997). Fossil ectomycorrhiza from the middle Eocene. American Journal of Botany, 84, 410–412. 10.2307/2446014 21708594

[ece34499-bib-0057] Le Page, Y. , Oom, D. , Silva, J. M. N. , Jönsson, P. , & Pereira, J. M. C. (2010). Seasonality of vegetation fires as modified by human action: Observing the deviation from eco‐climatic fire regimes. Global Ecology & Biogeography, 19, 575–588. 10.1111/j.1466-8238.2010.00525.x

[ece34499-bib-0058] Ledgard, N. (2001). The spread of lodgepole pine (*Pinus contorta*, Dougl.) in New Zealand. Forest Ecology and Management, 141, 43–57. 10.1016/S0378-1127(00)00488-6

[ece34499-bib-0059] Ledgard, N. (2004). Wilding conifers–New Zealand history and research background. *Managing wilding conifers in New Zealand–present and future* Proceedings of a workshop held in conjunction with the annual general meeting of the NZ Plant Protection Society in Christchurch on August. 1–25.

[ece34499-bib-0060] Lidgard, S. , & Crane, P. R. (1988). Quantitative analysis of the angiosperm radiation. Nature, 331, 344–346. 10.1038/331344a0

[ece34499-bib-0061] Lodhi, M. A. , & Killingbeck, K. T. (1982). Effects of pine‐produced chemicals on selected understory species in a *Pinus ponderosa* community. Journal of Chemical Ecology, 8, 275–283. 10.1007/BF00984023 24414602

[ece34499-bib-0062] López, G. G. , Kamiya, K. , & Harada, K. (2002). Phylogenetic relationships of Diploxylon pines (subgenus *Pinus*) based on plastid sequence data. International Journal of Plant Sciences, 163, 737–747.

[ece34499-bib-0063] Loudermilk, E. L. , Hiers, K. , Pokswinski, S. , O'Brien, J. , Barnett, A. , & Mitchell, R. J. (2016). The path back: Oaks (*Quercus* spp.) facilitate longleaf pine (*Pinus palustris*) seedling establishment in xeric sites. Ecosphere, 7(6), e01361 10.1002/ecs2.1361

[ece34499-bib-0064] Lubetkin, K. C. , Westerling, A. L. , & Kueppers, L. M. (2017). Climate and landscape drive the pace and pattern of conifer encroachment into subalpine meadows. Ecological Applications, 27, 1876–1886. 10.1002/eap.1574 28482135

[ece34499-bib-0065] Macdonald, G. M. , Cwynar, L. C. , & Whitlock, C. (1998). The late quaternary dynamic of pines in northern North America In Richardson D. M. (Ed.), Ecology and biogeography of Pinus (pp. 122–136). Cambridge, UK: Cambridge University Press.

[ece34499-bib-0066] Malloch, D. W. , Pirozynski, K. A. , & Raven, P. H. (1980). Ecological and evolutionary significance of mycorrhizal symbioses in vascular plants (a review). Proceedings of the National Academy of Sciences of the United States of America, 77, 2113–2118. 10.1073/pnas.77.4.2113 16592806 PMC348662

[ece34499-bib-0067] Mandle, L. , Bufford, J. L. , Schmidt, I. B. , & Daehler, C. C. (2011). Woody exotic plant invasions and fire: Reciprocal impacts and consequences for native ecosystems. Biological Invasions, 13, 1815–1827. 10.1007/s10530-011-0001-3

[ece34499-bib-0068] Matheny, P. B. , Aime, M. C. , Bougher, N. L. , Desjardin, D. E. , Horak, E. , … Hibbet, D. S. (2008). Out of the Palaeotropics? Historical biogeography and diversification of the cosmopolitan ectomycorrhizal mushroom family Inocybaceae. Journal of Biogeography, 36, 577–592. 10.1111/j.1365-2699.2008.02055.x

[ece34499-bib-0069] McGregor, K. F. , Watt, M. S. , Hulme, P. E. , & Duncan, R. P. (2012). What determines pine naturalization: Species traits, climate suitability or forestry use? Diversity and Distributions, 18, 1013–1023. 10.1111/j.1472-4642.2012.00942.x

[ece34499-bib-0070] Means, D. B. (1996). Longleaf pine forests, going, going… In Davis M. (Ed.), Eastern old‐growth forests (pp. 210–228). Washington, DC: Island Press.

[ece34499-bib-0071] Metlen, K. L. , & Callaway, R. M. (2015). Native North American pine attenuates the competitive effects of a European invader on native grasses. Biological Invasions, 17, 1227–1237. 10.1007/s10530-014-0790-2

[ece34499-bib-0073] Mirov, N. T. (1967). The genus Pinus. New York, NY: Ronald Press.

[ece34499-bib-0075] Morales‐Molino, C. , Tinner, W. , Garcia‐Anton, M. , & Colombaroli, D. (2017). The historical demise of *Pinus nigra* forests in the Northern Iberian plateau (south‐western Europe). Journal of Ecology, 105, 634–646. 10.1111/1365-2745.12702

[ece34499-bib-0076] Moreira, X. , Zas, R. , & Sampedro, L. (2013). Additive genetic variation in resistance traits of an exotic pine species: Little evidence for constraints on evolution of resistance against native herbivores. Heredity, 110, 449–456. 10.1038/hdy.2012.108 23232833 PMC3630812

[ece34499-bib-0077] Nasca, L. B. Z. , Relva, M. A. , & Núñez, M. A. (2018). Ungulates can control tree invasions: Experimental evidence from nonnative conifers and sheep herbivory. Biological invasions, 20, 583–591. 10.1007/s10530-017-1558-2

[ece34499-bib-0209] Näsholm, T. , Ekblad, A. , Nordin, A. , Giesler, R. , Hogberg, M. , & Hogberg, P. (1998). Boreal forest plants take up organic nitrogen. Nature, 392, 914–916. 10.1038/31921

[ece34499-bib-0078] Norris, J. R. , Betancourt, J. L. , & Jackson, S. T. (2016). Late Holocene expansion of ponderosa pine (*Pinus ponderosa*) in the Central Rocky Mountains, USA. Journal of Biogeography, 43, 778–790. 10.1111/jbi.12670

[ece34499-bib-0079] Northup, R. R. , Dahlgren, R. A. , Aide, T. M. , & Zimmerman, J. K. (1999). Effect of polyphenols on nutrient cycling and implications for community structure In Inderjit , Dakshini K. M. M. & Foy C. L. (Eds.), Principles and practices in plant ecology: Allelochemical interactions (pp. 255–266). Boca Raton, FL: CRC Press.

[ece34499-bib-0080] Northup, R. R. , Yu, Z. , Dahlgren, R. A. , & Vogt, K. A. (1995). Polyphenol control of nitrogen release from pine litter. Nature, 377, 227–229. 10.1038/377227a0

[ece34499-bib-0081] Nuñez, M. A. , Chiuffo, M. C. , Torres, A. , Paul, T. , Dimarco, R. D. , Raal, P. , … Richardson, D. M. (2017). Ecology and management of invasive Pinaceae around the world: Progress and challenges. Biological Invasions, 19, 3099–3120. 10.1007/s10530-017-1483-4

[ece34499-bib-0082] Nuñez, M. A. , Hayward, J. , Horton, T. R. , Amico, G. C. , Dimarco, R. D. , Barrios‐Garcia, M. N. , & Simberloff, D. (2013). Exotic mammals disperse exotic fungi that promote invasion by exotic trees. PLoS One, 8(6), e66832 10.1371/journal.pone.0066832 23826154 PMC3691272

[ece34499-bib-0083] Nuñez, M. A. , Horton, T. R. , & Simberloff, D. (2009). Lack of belowground mutualisms hinders Pinaceae invasions. Ecology, 90, 2352–2359. 10.1890/08-2139.1 19769113

[ece34499-bib-0084] Nuñez, M. A. , & Medley, K. A. (2011). Pine invasions: Climate predicts invasion success; something else predicts failure. Diversity and Distributions, 17, 703–713. 10.1111/j.1472-4642.2011.00772.x

[ece34499-bib-0085] Nuñez, M. A. , & Raffaele, E. (2007). Afforestation causes changes in post‐fire regeneration in native shrubland communities of northwestern Patagonia, Argentina. Journal of Vegetation Science, 18, 827–834. 10.1111/j.1654-1103.2007.tb02599.x

[ece34499-bib-0086] Nuñez, M. A. , Simberloff, D. , & Relva, M. A. (2008). Seed predation as a barrier to alien conifer invasions. Biological Invasions, 10, 1389–1398. 10.1007/s10530-007-9214-x

[ece34499-bib-0087] Paritsis, J. , Landesmann, J. , Kitzberger, T. , Tiribelli, F. , Sasal, Y. , Quintero, C. , … Nuñez, M. (2018). Pine plantations and invasion alter fuel structure and potential fire behavior in a Patagonian forest‐steppe ecotone. Forests, 9, 117 10.3390/f9030117

[ece34499-bib-0088] Pauchard, A. , Garcia, R. , Zalba, S. , Sarasola, M. , Zenni, R. , Ziller, S. , & Nuñez, M. A. (2015). Pine invasions in South America: Reducing their ecological impacts through active management In Canning‐Clode J. (Ed.), Biological invasions in changing ecosystems: Vectors, ecological impacts, management and predictions (pp. 318–342). Warsaw, Poland: De Gruyter Open Ltd.

[ece34499-bib-0089] Pauchard, A. , Milbau, A. , Albihn, A. , Alexander, J. , Burgess, T. , Daehler, C. , … Kueffer, C. (2016). Non‐native and native organisms moving into high elevation and high latitude ecosystems in an era of climate change: New challenges for ecology and conservation. Biological Invasions, 18, 345–353. 10.1007/s10530-015-1025-x

[ece34499-bib-0091] Peh, K. S.‐H. , Lewis, S. L. , & Lloyd, J. (2011). Mechanisms of monodominance in diverse tropical tree‐dominated systems. Journal of Ecology, 99, 891–898. 10.1111/j.1365-2745.2011.01827.x

[ece34499-bib-0092] Platt, W. J. , Evans, G. W. , & Rathbun, S. L. (1988). The population dynamics of a long‐lived conifer (*Pinus palustris*). American Naturalist, 131, 491–525.

[ece34499-bib-0093] Prévosto, B. , Hill, D. R. C. , & Coquillard, P. (2003). Individual‐based modelling of *Pinus sylvestris* invasion after grazing abandonment in the French Massif Central. Plant Ecology, 168, 121–137. 10.1023/A:1024404214782

[ece34499-bib-0094] Procheş, J.R.U. , Wilson, J. R. , Richardson, D. M. , & Rejmánek, M. (2012). Native and naturalized range size in *Pinus*: Relative importance of biogeography, introduction effort and species traits. Global Ecology & Biogeography, 21, 513–523. 10.1111/j.1466-8238.2011.00703.x

[ece34499-bib-0095] Raffaele, E. , Nuñez, M. A. , Enestron, J. , & Blackhall, M. (2016). Fire as mediator of pine invasion: Evidence from Patagonia, Argentina. Biological Invasions, 18, 597–601. 10.1007/s10530-015-1038-5

[ece34499-bib-0096] Read, D. J. , & Perez‐Moreno, J. (2003). Mycorrhizas and nutrient cycling in ecosystem – A journey towards relevance. New Phytologist, 157, 475–492. 10.1046/j.1469-8137.2003.00704.x 33873410

[ece34499-bib-0097] Rejmánek, M. , & Richardson, D. M. (1996). What attributes make some plant species more invasive? Ecology, 77, 1655–1661.

[ece34499-bib-0098] Rejmánek, M. , & Richardson, D. M. (2013). Trees and shrubs as invasive alien species – 2013 update of the global database. Diversity and Distributions, 19, 1093–1094. 10.1111/ddi.12075

[ece34499-bib-0099] Richardson D. M. (Ed.) (1998). Ecology and biogeography of Pinus. Cambridge, UK: Cambridge University Press.

[ece34499-bib-0100] Richardson, D. M. (2006). *Pinus*: A model group for unlocking the secrets of alien plant invasions? Preslia, 78, 375–388.

[ece34499-bib-0101] Richardson, D. M. , & Bond, W. J. (1991). Determinants of plant distribution: Evidence from pine invasions. American Naturalist, 137, 639–668.

[ece34499-bib-0102] Richardson, D. M. , & Brown, P. J. (1986). Invasion of mesic mountain fynbos by *Pinus radiata* . South African Journal of Botany, 52, 529–536. 10.1016/S0254-6299(16)31486-7

[ece34499-bib-0103] Richardson, D. M. , & Cowling, R. M. (1992). Why is mountain fynbos invasible and which species invade? In Van Wilgen B. W., Richardson D. M., Kruger F. J., & van Hensbergen H. J. (Eds.), Fire in South African mountain fynbos (pp. 161–181). Berlin, Germany: Springer‐Verlag.

[ece34499-bib-0104] Richardson, D. M. , Cowling, R. M. , & Le Maitre, D. C. (1990). Assessing the risk of invasive success in *Pinus* and *Banksia* in South African mountain fynbos. Journal of Vegetation Science, 1, 629–642. 10.2307/3235569

[ece34499-bib-0106] Richardson, D. M. , & Rejmánek, M. (2004). Invasive conifers: A global survey and predictive framework. Diversity and Distributions, 10, 321–331. 10.1111/j.1366-9516.2004.00096.x

[ece34499-bib-0107] Richardson, D. M. , Rundel, P. W. , Jackson, S. T. , Teskey, R. O. , Aronson, J. , Bytnerowicz, A. , … Procheş, S. (2007). Human impacts in pine forests: Past, present and future. Annual Reviews of Ecology, Evolution, and Systematics, 38, 275–297.

[ece34499-bib-0108] Richardson, D. M. , van Wilgen, B. W. , & Nuñez, M. (2008). Alien conifer invasions in South America – Short fuse burning? Biological Invasions, 10, 573–577. 10.1007/s10530-007-9140-y

[ece34499-bib-0109] Richardson, D. M. , Williams, P. A. , & Hobbs, R. J. (1994). Pine invasions in the Southern Hemisphere: Determinants of spread and invadability. Journal of Biogeography, 21, 511–527.

[ece34499-bib-0111] Rouget, M. , Richardson, D. M. , Milton, S. J. , & Polakow, D. (2001). Invasion dynamics of four alien *Pinus* species in a highly fragmented semi‐arid shrubland in South Africa. Plant Ecology, 152, 79–92. 10.1023/A:1011412427075

[ece34499-bib-0112] Rundel, P. W. , Dickie, I. E. , & Richardson, D. M. (2014). Tree invasions into treeless areas: Mechanisms and ecosystem processes. Biological Invasions, 16, 663–675. 10.1007/s10530-013-0614-9

[ece34499-bib-0113] Sarasola, M. , Rusch, V. , Schlichter, T. , & Ghersa, C. (2006). Tree conifers invasion in steppe areas and *Austrocedus chilensis* forests in NW Patagonia. Ecologia Austral, 16, 143–156.

[ece34499-bib-0114] Schwilk, D. W. , & Ackerly, D. D. (2001). Flammability and serotiny as strategies: Correlated evolution in pines. Oikos, 94, 326–336.

[ece34499-bib-0116] Simberloff, D. , Nuñez, M. A. , Ledgard, N. J. , Pauchard, A. , Richardson, D. M. , Sarasola, M. , … Ziller, S. R. (2010). Spread and impact of introduced conifers in South America: Lessons from other southern hemisphere regions. Austral Ecology, 35, 489–504. 10.1111/j.1442-9993.2009.02058.x

[ece34499-bib-0117] Simberloff, D. , & von Holle, B. (1999). Positive interactions of nonindigenous species: Invasional meltdown? Biological Invasions, 1, 21–32. 10.1023/A:1010086329619

[ece34499-bib-0118] Singh, J. S. , & Singh, S. P. (1992). Forests of Himalaya. India: Gyanodaya Prakashan, Nainital, India.

[ece34499-bib-0119] Singh, S. P. , Zobel, D. B. , Garkoti, S. C. , Tewari, A. , & Negi, C. M. S. (2006). Patterns in water relations of central Himalayan trees. Tropical Ecology, 47, 159–182.

[ece34499-bib-0120] Stralberg, D. , Wang, X. , Parisien, M. A. , Robinne, F. N. , Solymos, P. , Mahon, C. L. , … Bayne, E. M. (2018). Wildfire‐mediated vegetation change in boreal forests of Alberta, Canada. Ecosphere, 9, e02156 10.1002/ecs2.2156

[ece34499-bib-0121] Strauss, S. H. , & Ledig, F. T. (1985). Seedling architecture and life history evolution in pines. American Naturalist, 125, 702–715.

[ece34499-bib-0122] Strimbeck, G. R. , Schaberg, P. G. , Fossdal, C. G. , Schröder, W. P. , & Kjellsen, T. D. (2015). Extreme low temperature tolerance in woody plants. Frontiers in Plant Science, 6, 884 10.3389/fpls.2015.00884 26539202 PMC4609829

[ece34499-bib-0123] Stuchlik, L. (1994). Some late Pliocene and early Pleistocene pollen profiles from Poland In Boulter M. C. & Fisher H. C. (Eds.), Cenozoic plants and climates of the arctic (pp. 371–382). NATO ASI Series (Series I: Global Environmental Change), Vol. 27 Berlin, Germany: Springer.

[ece34499-bib-0124] Suseela, V. , & Tharayil, N. (2017). Decoupling the direct and indirect effects of climate on plant litter decomposition: Accounting for stress induced modifications in plant chemistry. Global Change Biology, 24, 1428–1451. 10.1111/gcb.13923 28986956

[ece34499-bib-0125] Taylor, K. T. , Maxwell, B. , McWerthy, D. B. , Pauchard, A. , Nuñez, M. A. , & Whitlock, C. (2017). *Pinus contorta* invasions increase wildfire fuel loads and may create a positive feedback with fire. Ecology, 98, 678–687. 10.1002/ecy.1673 27935641

[ece34499-bib-0126] Taylor, K. T. , Maxwell, B. D. , Pauchard, A. , Nuñez, M. A. , Peltzer, D. A. , Terwei, A. , & Rew, L. J. (2016a). Drivers of plant invasion vary globally: Evidence from pine invasions within six ecoregions. Global Ecology and Biogeography, 25, 96–106. 10.1111/geb.12391

[ece34499-bib-0127] Taylor, K. T. , Maxwell, B. , Pauchard, A. , Nuñez, M. A. , & Rew, L. (2016b). Native versus non‐native invasions: Similarities and differences in the biodiversity impacts of *Pinus contorta* in introduced and native ranges. Diversity and Distributions, 22, 578–588. 10.1111/ddi.12419

[ece34499-bib-0128] Thonicke, K. , Venevsky, S. , Sitch, S. , & Cramer, W. (2001). The role of fire disturbance for global vegetation dynamics: Coupling fire into a dynamic global vegetation model. Global Ecology and Biogeography, 10, 661–677. 10.1046/j.1466-822X.2001.00175.x

[ece34499-bib-0129] Tomback, D. F. (1982). Dispersal of whitebark pine seeds by Clark's Nutcracker: A mutualism hypothesis. Journal of Animal Ecology, 51, 451–467.

[ece34499-bib-0130] Tomback, D. F. , & Linhart, Y. B. (1990). The evolution of bird‐dispersed pines. Evolutionary Ecology, 4, 185–219. 10.1007/BF02214330

[ece34499-bib-0131] Valladares, F. , & Niinemets, Ü. (2008). Shade tolerance, a key plant feature of complex nature and consequences. Annual Review of Ecology, Evolution, and Systematics, 39, 237–257. 10.1146/annurev.ecolsys.39.110707.173506

[ece34499-bib-0132] Van Wilgen, B. W. , & Richardson, D. M. (2012). Three centuries of managing introduced conifers in South Africa: Benefits, impacts, changing perceptions and conflict resolution. Journal of Environmental Management, 106, 56–68. 10.1016/j.jenvman.2012.03.052 22562012

[ece34499-bib-0133] Vander Wall, S. B. , & Balda, R. P. (1977). Coadaptations of the Clark's Nutcracker and the pinon pine for efficient seed harvest and dispersal. Ecological Monographs, 47, 89–111.

[ece34499-bib-0134] Wan, T. , Liu, Z. M. , Li, L. F. , Leitch, A. R. , Leitch, I. J. , Lohaus, R. , … Wang, X. M. (2018). A genome for gnetophytes and early evolution of seed plants. Nature Plants, 4, 82–89. 10.1038/s41477-017-0097-2 29379155

[ece34499-bib-0136] Waring, R. H. , & Franklin, J. F. (1979). Evergreen coniferous forests of the Pacific Northwest. Science, 204, 1380–1386. 10.1126/science.204.4400.1380 17814182

[ece34499-bib-0137] Waring, R. H. , & Schlesinger, W. H. (1985). Forest ecosystems. Orlando, FL: Academic Press.

[ece34499-bib-0138] Westerling, A. L. , Hidalgo, H. G. , Cayan, D. R. , & Swetnam, T. W. (2006). Warming and earlier spring increase western U.S. forest wildfire activity. Science, 313, 940–943. 10.1126/science.1128834 16825536

[ece34499-bib-0139] Westoby, M. , Falster, D. S. , Moles, A. T. , Vesk, P. A. , & Wright, I. J. (2002). Plant ecological strategies: Some leading dimensions of variation between species. Annual Review of Ecology and Systematics, 33, 125–159. 10.1146/annurev.ecolsys.33.010802.150452

[ece34499-bib-0210] Willis, K. J. , & McElwain, J. C. (2002). The evolution of plants. Oxford, UK: Oxford University Press.

[ece34499-bib-0140] Wood, J. R. , Dickie, I. A. , Moeller, H. V. , Peltzer, D. A. , Bonner, K. I. , Rattray, G. , & Wilmshurst, J. M. (2015). Novel interactions between non‐native mammals and fungi facilitate establishment of invasive pines. Journal of Ecology, 103, 121–129. 10.1111/1365-2745.12345

[ece34499-bib-0141] Wright, S. J. , Kitajima, K. , Kraft, N. J. B. , Reich, P. B. , Wright, I. J. , Bunker, D. E. , … Zanne, A. E. (2010). Functional traits and the growth–mortality trade‐off in tropical trees. Ecology, 91, 3664–3674. 10.1890/09-2335.1 21302837

[ece34499-bib-0142] Yeaton, R. I. (1978). Some ecological aspects of reproduction in the genus *Pinus* L. Bulletin of the Torrey Botanical Club, 105, 306–311.

[ece34499-bib-0143] Zas, R. , Moreira, X. , & Sampedro, L. (2011). Tolerance and induced resistance in a native and an exotic pine species: Relevant traits for invasion ecology. Journal of Ecology, 99, 1316–1326. 10.1111/j.1365-2745.2011.01872.x

[ece34499-bib-0144] Zenni, R. D. , & Simberloff, D. (2013). Number of source populations as a potential driver of pine invasions in Brazil. Biological Invasions, 15, 1623–1639. 10.1007/s10530-012-0397-4

[ece34499-bib-0145] Zong, C. , Wauters, L. A. , Van Dongen, S. , Mari, V. , Romeo, C. , Martinoli, A. , … Tosi, G. (2010). Annual variation in predation and dispersal of Arolla pine (*Pinus cembra* L.) seeds by Eurasian red squirrels and other seed‐eaters. Forest Ecology and Management, 260, 587–594. 10.1016/j.foreco.2010.05.014

